# Supplementary material for: Morphological changes after cranial fractionated photon radiotherapy: Localized loss of white matter and grey matter volume with increasing dose
Source: Clin Transl Radiat Oncol. 2021 Aug 29;31:14–20. doi: 10.1016/j.ctro.2021.08.010 (PMC8416633; doi:10.1016/j.ctro.2021.08.010)
Supplement: Supplementary data 2 [file mmc2.docx]

| **Supplementary table 1** Cortical regions in which more than 40% of voxels show dose-dependent volume loss in deformation-based morphometry | | | |
| --- | --- | --- | --- |
| **Region name** | **Affected voxels within area (%)** | **Relative volume change (%/30 Gy)** | **p-value** |
| Left Frontal Operculum | 67.7 | 15.0 | <0.01 |
| Left Anterior Insula | 51.6 | 10.5 | <0.01 |
| Left Superior Temporal Gyrus | 51.4 | 5.3 | <0.01 |
| Left Middle Occipital Gyrus | 46.7 | 10.8 | <0.01 |
| Left Inferior Frontal Angular Gyrus | 45.1 | 15.3 | <0.01 |
| Left Supplementary Motor Cortex | 44.5 | 9.3 | <0.01 |
| Left Middle Temporal Gyrus | 43.9 | 8.4 | <0.01 |
| Left Planum Temporale | 43.8 | 7.0 | <0.01 |
| Left Inferior Occipital Gyrus | 41.4 | 10.5 | <0.01 |
| Left Precuneus | 40.0 | 10.3 | <0.01 |
